# Assessing the Co‐Occurrence of European Pine Marten (*Martes martes*) With Humans and Domestic Cats on a Mediterranean Island

**DOI:** 10.1002/ece3.70651

**Published:** 2024-11-29

**Authors:** Emiliano Manzo, Fabiola Iannarilli, Filippo Dell'Agnello, Paola Bartolommei, Andrea Bonacchi, Stefania Gasperini, Roberto Cozzolino

**Affiliations:** ^1^ Fondazione Ethoikos, Convento dell'Osservanza S.N.C. Radicondoli Siena Italy; ^2^ Department of Migration Max Planck Institute of Animal Behavior Konstanz Germany

**Keywords:** camera trapping, cat coexistence, Elba Island, human disturbance, island ecosystem, species interactions

## Abstract

Anthropogenic activities often lead to changes in the distribution and behavior of wild species. The mere presence of humans and free‐roaming domestic cats ( *Felis catus* ) can affect wildlife communities; however, responses to these disturbances might not be ubiquitous and may vary with local conditions. We investigated European pine marten's ( *Martes martes* ) distribution on Elba Island, Italy, where the species is the only wild carnivore. In this system, pine martens act as the top predator, and human presence is mostly driven by seasonal tourism. We evaluated (1) pine marten's occurrence in relation to vegetation type and elevation and the potential effects of proximity to settlements, (2) whether pine marten's distribution was associated with the co‐occurrence of humans and domestic cats, and, if so, (3) whether these co‐occurrence patterns were associated with proximity to anthropogenic infrastructures. Additionally, we explored similarities in activity patterns between pine marten and the other two species. We collected camera‐trap data at 77 locations throughout Elba Island in February–July 2020. Using single‐season multistate occupancy models, we found evidence that pine martens' occupancy was generally high across all vegetation types and elevation, and proximity to settlements was only weakly associated with the species occurrence. Contrary to expectations, we found no evidence of an association between pine martens' distribution and the presence of either humans or free‐roaming domestic cats on Elba Island. Opposing activity patterns might have facilitated pine martens' co‐existence with humans, with pine martens being active at ground level almost exclusively during nighttime. On the contrary, cats and pine martens showed similar activity patterns, and further studies are needed to define the co‐existence mechanisms. These findings have important management implications and suggest that response to direct and indirect anthropogenic pressures can be highly context‐dependent and mediated by the availability of resources and competition mechanisms.

## Graphical abstract

We explored the co‐occurrence of pine marten, *Martes martes* , and humans and domestic cats on Elba Island, in the Mediterranean Sea. Pine marten occupancy was not affected by the occurrence of humans and domestic cats, despite their widespread presence. Opposite patterns of activity might explain the co‐occurrence of martens and humans, whereas more research is needed to explore the mechanisms behind the co‐occurrence of martens and cats.

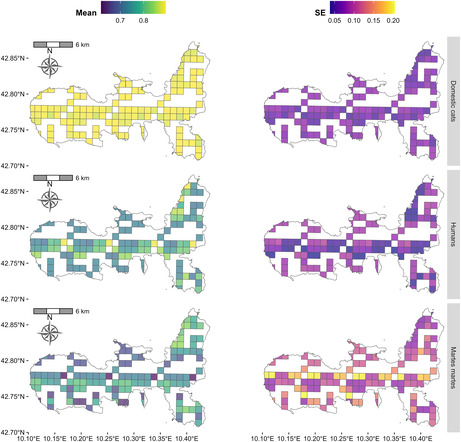

## 1 Introduction

The study of the impact of anthropogenic activities on wildlife has so far primarily focused on assessing the effect of large‐scale—often permanent and irreversible—changes such as land use modification, habitat fragmentation, increased traffic on roads, and the introduction of alien species (Ceballos and Ehrlich 2002; Crooks, Scott, and Van Vuren 2001; Czech, Krausman, and Devers 2000; Gaynor et al. 2018; Trouwborst and Somsen 2020). Technological advancements (e.g., in telemetry and camera trapping) have recently enabled researchers to explore the effect of human activities at unprecedented spatiotemporal resolutions. New evidence shows that anthropogenic disturbance might also act at local and instantaneous scales and that even the mere presence of humans and free‐ranging domestic species could lead to behavioral changes in wildlife (Higginbottom, Northrope, and Green 2001; Higham and Shelton 2011; Korhonen, Jauhiainen, and Rekila 2002; Loss, Will, and Marra 2013; Young et al. 2011).

The presence of humans in natural environments might exert a significant negative influence on wild species, even when such presence is limited in time, as is often the case for many nature‐based tourism‐related activities (Hammitt and Cole 1998; Kays et al. 2016; Larson et al. 2016). The presence of people might instill fear in wild species, leading to behavioral modifications such as movement toward suboptimal habitats, reduced activity levels, and restricted movements (Corradini et al. 2021; Frid and Lawrence 2002; Gaynor et al. 2018; Marion et al. 2020; Markovchick‐Nicholls et al. 2008). Despite the increasing recognition of outdoor recreational activities as a potential threat to endangered species, it is important to acknowledge that the impact might not be universally negative (Tucker et al. 2023). Certain wild species such as herbivore mammals may exhibit adaptive responses that result in beneficial effects derived from tourism‐related activities; Muhly et al. (2011), for example, showed that some ungulates might be attracted to highly trafficked trails because of the potential protection from predators resulting from the presence of people along those trails (i.e., human shield effect). People often seek nature for its beneficial effects on their mental and physical well‐being (Winter et al. 2019). Nature‐based recreational activities, such as hiking and biking, are common ways in which humans spend time in nature. Outdoor recreation, including hiking and biking, emerges as a key component of human well‐being and significantly contributes to the livelihood of local communities. Nonetheless, given the consequences on wildlife, there is an urgent need to strike a balance between the occurrence of these recreational activities and their potential impacts on wildlife communities in touristic settings.

The presence of species commonly associated with humans, such as dogs *Canis lupus familiaris* and domestic cats *Felis catus* , might also affect wildlife (Hughes and MacDonald 2013; Lessa et al. 2016; Medina et al. 2011). In particular, free‐roaming cats, that is, owned or unowned cats that are allowed to roam freely for their whole life or part of it, are among the most ubiquitous and environmentally damaging invasive predators, and their presence raises concerns for wildlife conservation (Trouwborst, McCormack, and Martínez Camacho 2020). Cats significantly impact biodiversity through predation, fear effects, competition, disease, and hybridization (Trouwborst, McCormack, and Martínez Camacho 2020). In both rural and urban environments, cats have historically been allowed to roam freely, either due to their role as mousers in rural areas or as companion animals allowed to explore the outside for certain periods (Beutel et al. 2017; Sims et al. 2008). As such, cats often spend long periods exploring the outdoors and functionally act as predators or competitors of many wild species, representing one of the main threats to global biodiversity (Loss, Will, and Marra 2013; Mori et al. 2019).

Here, we assessed whether the presence of domestic cats and humans was associated with the distribution and activity pattern of a medium‐sized carnivore, the European pine marten *Martes martes* , on Elba Island, an insular ecosystem in the Mediterranean Sea. On Elba Island, pine martens occur in the absence of other wild carnivorous competitors; in this context, domestic cats might serve as the primary competitor of the species and, consequently, be associated with its distribution and time of activity. Concurrently, the scenario of Elba Island also offers the opportunity to explore the effect of the presence of humans on pine martens. The number of people on the island is highly variable throughout the year and is mainly driven by tourism, which is the main revenue stream for most of the thirty thousand permanent residents of Elba Island (ISTAT 2020). More than 1 million people visit the island each year, primarily during the warmest months (May–September; Regione Toscana 2021). Many of these visitors take advantage of the extensive network of hiking and biking trails that encompass the island. As such, the resulting human presence in the landscape could be a key driver of the distribution and temporal activity of pine martens on Elba Island.

Using Elba Island as a natural experimental setting, we aimed to assess natural and anthropogenic factors correlated with pine marten's distribution. In particular, we focused on the spatiotemporal patterns of overlap of pine martens, humans, and cats on the island. We first focused on exploring pine martens' occurrence independently from the other two species and in response to vegetation type, elevation, and distance to settlements. We then assessed the separation of pine marten's niche in relation to those of humans and cats along the spatial axis and investigated whether pine marten's distribution was related to the co‐occurrence of humans and domestic cats, and, if so, whether these co‐occurrence patterns changed with proximity to anthropogenic infrastructures (i.e., distances from roads and settlements). We also focused on temporal niche overlap and assessed the overlap in ground‐level activity between pine martens and each of the other two species. Given the lack of natural competitors on Elba Island, we expected that pine marten would occupy several of the environments available on the island. Based on previous knowledge (Mori et al. 2021), we expected that the presence of humans would correspond to a decrease in the probability of occupancy of pine marten in this environment. We also expected similar patterns for the presence of cats. Free‐roaming cats might be the most direct competitor of pine marten on the island and lead to a decrease in occupancy and activity of this mustelid through direct and indirect effects. To test our hypotheses, we extracted the information about the pattern of site use of the three species from camera‐trap data collected at different sites across the whole island and ran a novel occupancy framework that allows us to directly model the co‐occurrence of two (or more) species (Rota et al. 2016). Using this approach, we tested the factors associated with these co‐occurrence patterns.

## 2 Methods

### 2.1 Study Area

**FIGURE 1:**
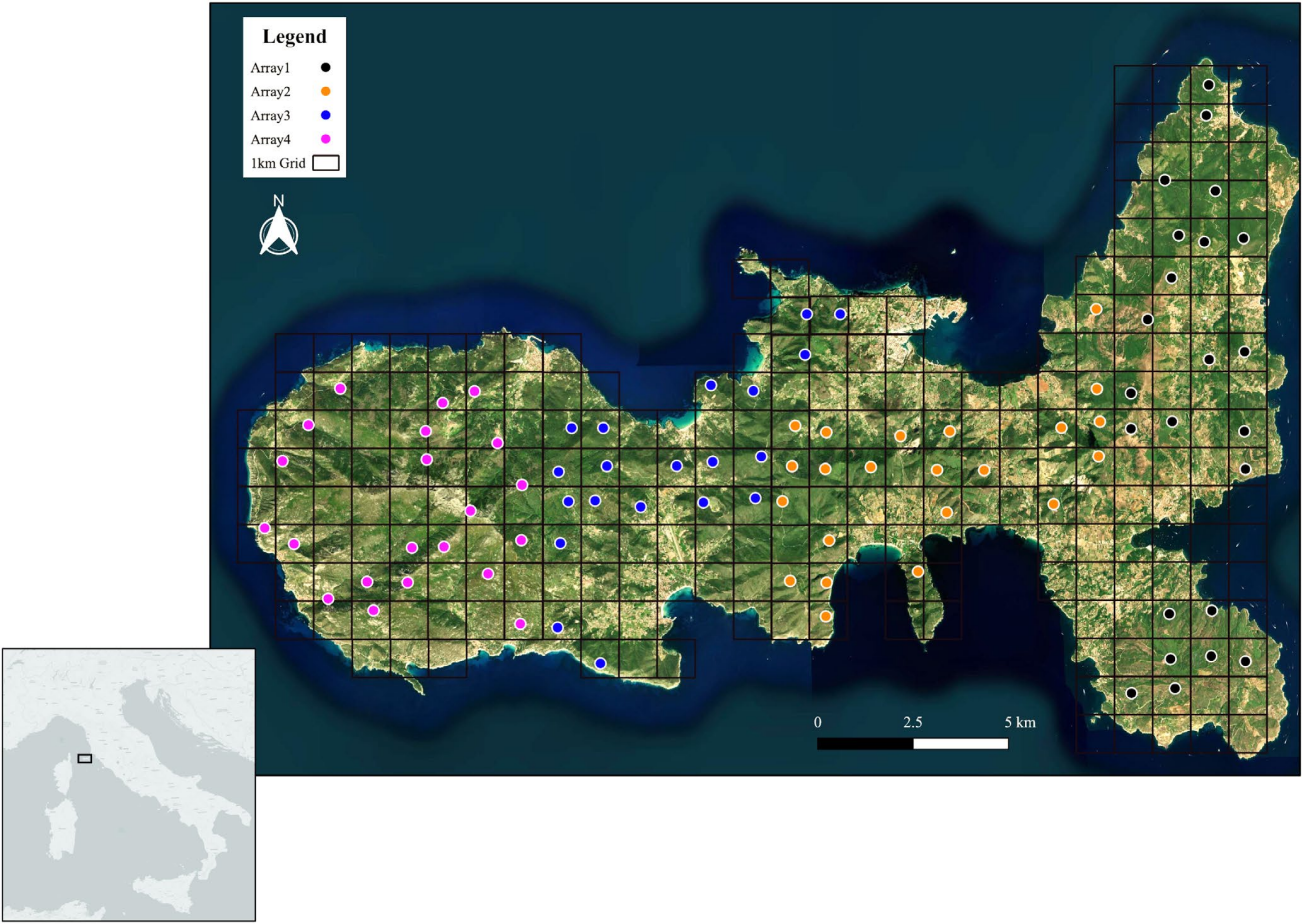
Map of Elba Island showing the 1 × 1 km cell grid and the locations sampled (dots). The grid was divided into four groups (i.e., arrays, color‐coded in the figure) and, within each group, 20–30 sites were selected. The arrays were sampled sequentially in February–July 2020. Each camera site was selected as close as possible to the centroid of a cell. In each location, a Bushnell Trophy cam HD Aggressor No Glow camera was deployed for at least 30 camera‐trap days.

This study was undertaken in Central Italy within the Tuscan Archipelago National Park (hereafter, TANP) and covered the whole Elba Island (42°47′12″ N, 10°16′28″ E; Figure 1). Elba Island is the largest island of the Tuscan Archipelago with a total area of 223 km^2^. The climate is Mediterranean with a mean annual temperature of 16.5°C (min. 10°C in January, max. 24.5°C in July) and mean yearly rainfall of 595 mm (min. 13 mm in July, max. 86 mm in November). More than half of the island (127.4 km^2^) was designated as a National Park in 1996. The island is characterized by high geomorphological heterogeneity and an altitude ranging from sea level up to 1019 m a.s.l. (Monte Capanne). This environmental heterogeneity leads to the establishment of three distinct bioclimatic belts and a large vegetation diversity (Foggi et al. 2006), which includes holm oak *Quercus ilex* L. woods, low Mediterranean maquis characterized by *Salvia rosmarinus* Spenn., and *Lavandula stoechas* L. and *Cistus* spp., high Mediterranean maquis—with vegetation higher than 1 m, characterized by strawberry trees *Arbutus unedo* L. and tree heath *Erica arborea* L.—, coniferous plantations, and chestnut *Castanea sativa* Mill. groves (Foggi et al. 2006; Meriggi et al. 2016). Settlements and farming activities occur outside the TANP's borders, with an abundance of orchards and grape vines, and a network of paved roads connecting villages. Several mammals are present within the park including rodents and shrews, wild boars *Sus scrofa* , European mouflons *Ovis aries* , Apennine hares *Lepus corsicanus* , and European brown hares *Lepus europaeus* (Greco et al. 2021). The European pine marten *Martes martes* is the only wild carnivore species present on Elba Island (Greco et al. 2021; Loy et al. 2019).

### 2.2 Data Collection

Camera locations were selected by partitioning the island into 1 × 1 km cells using QGIS (QGIS Development Team 2019). The whole grid was divided into four camera‐trap arrays (Figure 1). Each array was composed of 20–30 randomly selected cells where a camera trap was placed, at a density of at least one camera per km^2^. Each array was sampled sequentially between February 1 and July 20, 2020. Camera traps were placed as close as possible to the centroid of each cell (Figure 1), at locations with a suitable tree for mounting the traps and where the area around the point was sufficiently open for the camera to have a clear view. In some cases, the dense Mediterranean vegetation on Elba Island did not allow for setting up cameras on the predefined centroid without causing pronounced damage to the environment; in other cases, the steep topography limited access to the predefined locations. The median displacement of camera stations from planned centroids was 165 m (range: 16–534 m). Cameras were deployed at each site for at least 30 consecutive days. At each camera station, one Bushnell Trophy Cam HD Aggressor No Glow camera (model 119877; Overland Park, Kansas, USA) was mounted on a homemade metal bracket and attached to trees or shrubs at a height of approximately 0–40 cm above the ground (depending on the ground slope) and secured with chains and padlocks to prevent theft. No baiting or attractants were used and cameras were powered by Lithium Ultimate Energizer batteries. Camera traps were set to record videos for 30 s, with a trigger interval of 60 s between two consecutive videos. At each site, we attached signs informing passers‐by about the study's purpose, requesting to leave the camera undisturbed, and providing contact information. Fieldwork activities and camera trapping were carried out with the authorization of the TANP no. 5106/2019.

### 2.3 Data Processing

All videos were reviewed by expert researchers and tagged using the open‐source photo management tool “digiKam” (https://www.digikam.org). We annotated the presence of pine martens, humans (walking and cycling), and domestic cats. Without access to ancillary information, we were not able to establish whether the cats detected in our camera‐trap videos were owned or unowned and to which degree humans controlled their behavior in terms of food provision, movement, and reproduction. Consequently, we can only assume these cats were allowed to roam freely for at least part of the day and that each of them fell somewhere along the gradient ranging from indoor–outdoor, to free‐ranging, and feral cats (sensus Crowley, Cecchetti, and McDonald 2019). Hereinafter, we will refer to these cats with the term free‐roaming domestic cats. Metadata and tags were extracted from the videos using the R package *camtrapR* (Niedballa et al. 2016). We organized the data in species‐specific detection histories using the *detectionHistory* function of the same package; we considered occasions as 1‐day long, with detection histories at each site starting on the first day of sampling at that site.

### 2.4 Covariates

Our model sets included both environmental (vegetation type, elevation) and anthropogenic (distance to settlement, distance to roads) covariates, along with variables that characterized the sampling process (sampling date, and whether COVID‐19 lockdown limitations to human movement were in place). Using QGIS (QGIS Development Team 2019) and starting from the island's vegetation map (Foggi et al. 2006), we aggregated the different vegetation classes into five categories: *Quercus ilex* forest, low and high Mediterranean maquis, coniferous forest, and other vegetation types. We then established the dominant vegetation type (*VegType*) in each cell by overlapping the location of the relative camera‐trap site with the resulting map showing these five vegetational categories. The final set of camera‐trap locations included 25 sites in *Quercus ilex* forests, 21 and 19 sites in low and high Mediterranean maquis, respectively, 8 in coniferous forest and 8 in other vegetation types (azonal vegetation and patches dominated by *C. sativa* and *Q. suber* ). Using QGIS, we also extracted the minimum distance of each location to settlements (*DistSettl*; data source: Corine Land Cover 10k Regione Toscana) and to paved roads (*DistRoads*; data source: Corine Land Cover 10k Regione Toscana) using the tool *Shortest line between*. We extracted *elevation* (in meters) at each location using package *rstoat* (Wilshire, Li, and Ranipeta 2021; data source: NASA Shuttle Radar Topography Mission [SRTM] 2013) in R (R Core Team 2022; version 4.2.1). We built a site‐by‐observation matrix reporting days of sampling as *Julian date*, and a matrix of the same dimensions to characterize the days of sampling as before/during/after COVID‐19 *lockdown* (0/1/2), with lockdown happening between March 9 and May 17, 2020. We did not include the distance of the camera‐trap locations to the nearest hiking trail as a covariate because all locations were relatively close to at least one trail, with distances ranging from 0.1 m to 432 m and an average distance of 60.5 m. Previous studies showed that distance to roads and distance to settlements are good descriptors of space used by humans and cats, respectively (Bird 2021; Morin et al. 2018; Odell and Knight 2001). Consequently, we decided to use these covariates to model the heterogeneity in the occupancy probability of humans and cats, respectively, across Elba Island (see “Occupancy modeling” section).

Prior to running any occupancy models, we tested for the level of correlation among the continuous covariates using Pearson's correlation; no pairs had correlation values higher than |0.60| (Figure S1 in Appendix S1). All numeric covariates were standardized (i.e., scaled and centered; mean = 0, standard deviation = 1) before running the analysis.

### 2.5 Statistical Analysis

#### 2.5.1 Occupancy Modeling

We applied multispecies occupancy models (Rota et al. 2016) to assess pine marten's distribution throughout Elba Island and quantify whether and how its distribution was associated with the co‐occurrence of humans or free‐roaming domestic cats. Hierarchical occupancy models estimate the probability that a certain species occupies a specific area while accounting for imperfect detection, that is, failing to detect the species even when present. The multispecies framework used in this analysis enables us to explore how the occurrence of a certain species (e.g., pine marten) varies along a certain gradient (e.g., an environmental variable) conditional on whether or not another species (e.g., human) is present, while still accounting for imperfect detection. Similar to the single‐species occupancy model (MacKenzie et al. 2002), the multispecies framework requires *t* repeated sampling at *n* sites randomly selected from the population of interest. Observations of species *s* at site *i* are organized in detections (*y*_*sit*_ = 1) and nondetections (*y*_*sit*_ = 0). Given *S* number of species, the latent (i.e., partially observed) occupancy state at site *i*, *z*_*i*_, consists of 2^s^ possible combinations. For example, when *S* = 2, *z*_*i*_ can take four possible states, *z*_*i*_ = ([00], [01], [10], [11]), where [00] represents the state in which both species are absent at site *i*, [01] represents the state in which the first species is absent but the second is present, and so on. *z*_*i*_ is defined as a multivariate Bernoulli random variable: *z*_*i*_ ~ MVB(*Ψ*_*i*_), where *Ψ*_*i*_ represents the probability of a certain state (e.g., *Ψ*_00*i*_) at site *i*. States are defined as first, second, third, up to *S*th‐order natural parameters based on the number of species present. For example, [10] and [01] correspond to the first‐order natural parameters ƒ_1_ (i.e., only the first species is present) and ƒ_2_ (i.e., only the second species is present), respectively, and [11] is the second‐order natural parameter ƒ_12_ (i.e., both species are present). Each natural parameter can be modeled as a function of covariates using the multinomial logit link (i.e., softmax) function. Consequently, we can model both single‐species occupancy (i.e., using first‐order natural parameters) and co‐occurrence of 2 up to *S* species (i.e., using higher‐order natural parameters); however, interpretation of relationships beyond the second order may be quite challenging and the models extremely data hungry. As such, we limited our modeling efforts to second‐order states. Applying the equations defined in Rota et al. (2016), the model combines the natural parameters to return the probability of each latent state (e.g., the probability of co‐occurrence of the two species as site *i*, *Ψ*_11*i*_). Setting all the natural parameters of order higher than 1 equal to zero assumes independence among the *S* species and returns the *marginal occupancy* (i.e., the occupancy across all possible states) of a certain species at the different sites. Modeling natural parameters higher than the first order allows one to estimate a species' *conditional occupancy*, that is how the occupancy of a species changes in response to the presence of another species and how this change varies along a certain variable.

Using this multispecies framework, we built alternative model structures to test our hypotheses. We followed a multistep approach. Keeping occupancy constant, we first modeled the probability of detection as a function of (1) the linear effect of *Julian date*, to test for linear increase or decrease in detection over time, (2) the linear and quadratic effect of *Julian date*, to also test for seasonal trends, (3) and *lockdown*, to test for changes associated with the differential human use of the trail system driven by the regulations that were set up during the initial phase of the COVID‐19 pandemic and that drastically limited the movement of people in Italy. We also ran a model with no covariates on detection (null model). We then retained the structure of the best‐supported detection model and tested two different competing hypotheses on factors associated with pine marten's marginal occupancy (i.e., independently of the presence/absence of the other two species; Table 1). We compared the support of two models, the first with pine marten's marginal occupancy as a function of vegetation type and elevation only (Mod0A), and the second where we also included distance to settlements (Mod0B); we made this comparison given the negative effects of urban areas on pine marten occupancy found by Mori et al. (2021). In models Mod0A and Mod0B (and throughout the rest of the occupancy analyses), humans' and cats' marginal occupancy probabilities were modeled as a function of distance to roads and distance to settlements, respectively, as these covariates well capture the patterns in space used by these two species in ecosystems comparable to Elba Island (Bird 2021; Morin et al. 2018; Odell and Knight 2001). Retaining the most supported model in this second set, we built three different model structures to describe three different concurrent hypotheses on pine marten's co‐occurrence with humans and cats (conditional occupancy): (1) pine martens' occupancy was *independent* of the presence of the other species (Mod1, equivalent to the best‐ranked model in the previous step); (2) pine martens' occupancy was *dependent* on the presence of the other species but *constant* (Mod2); and (3) pine martens' occupancy was *dependent* on the presence of the other species and the co‐occurrence of the two species *varied* along gradients (Mod3) defined by distance to roads (Mod3A), distance to settlements (Mod3B), the additive effect of distance to roads and distance to settlements (Mod3C), the interactive effect of distance to settlements and elevation (Mod3D) and distance to roads and elevation (Mod3E; Table 1). We chose to test the relative importance of these specific covariates as they are often cited as drivers of pine marten distribution. Several studies have shown that pine martens tend to preferentially select areas away from roads (Mod3A; Balestrieri et al. 2019; Virgós et al. 2012, and references therein) and human settlements (Mod3B; Balestrieri et al. 2019; Mori et al. 2021). We also decided to test for the interactive effect of elevation on the distance to roads and settlements, respectively, because of the sharp change in elevation (a change from 0 to 1019 m asl. in about a distance of ~5 km) that characterizes Elba Island. The ~30,000 inhabitants and their activities tend to be primarily concentrated along the coast and at lower altitudes, whereas the inland areas are left as natural or semi‐natural areas. We compared our alternative hypotheses using the Akaike Information Criterion, AIC (Burnham and Anderson 2002) and ran all occupancy models using the *occuMulti* function from package *unmarked* (Fiske and Chandler 2011). We mapped the estimated occupancy probability (mean and SE) of the three species considered across Elba Island based on the top‐ranked model for all the 1 × 1 km cells where the dominant vegetation type matched one of the categories included in the sampling.

**TABLE 1:** List of the alternative structures for the occupancy component of the models included in the final model set and the relative hypotheses tested.

	Description/hypothesis	Structure^a^
Mod1	Independent: Marten occupancy probability is *independent of the occurrence* of the other species	ƒ_12_ = ƒ_13_ = ƒ_23_ = 0
Mod2	Dep. Costant: Marten occupancy probability *depends on the occurrence* of the other species but their co‐occurrence pattern *does not vary* across gradients	ƒ_12_ = *β*_9_ ƒ_13_ = *β*_10_ ƒ_23_ = 0
Mod3	Dep. varying across gradients: Marten occupancy probability *depends on the occurrence* of the other species and their co‐occurrence *varies* across a gradient defined by…	
Mod3A	Dep. Road Dist.: …distance to roads	ƒ_12_ = *β*_11_ + *β*_12_*DistRoad ƒ_13_ = *β*_13_ + *β*_14_*DistRoad ƒ_23_ = 0
Mod3B	Dep. Settl. Dist.: …distance to settlements	ƒ_12_ = *β*_15_ + *β*_16_*DistSettl ƒ_13_ = *β*_17_ + *β*_18_*DistSettl ƒ_23_ = 0
Mod3C	Dep. Road + Settl. Dist.: …the additive effect of distance to roads and distance to settlements	ƒ_12_ = *β*_19_ + *β*_20_*DistRoad + *β*_21_*DistSettl ƒ_13_ = *β*_22_ + *β*_23_*DistRoad + *β*_25_*DistSettl ƒ_23_ = 0
Mod3D	Dep. Settl. Dist. × Ele: …the interaction of distance to settlements and elevation	ƒ_12_ = *β*_25_ + *β*_26_*Ele + *β*_27_*DistSettl + *β*_28_*Ele*DistSettl ƒ_13_ = *β*_29_ + *β*_30_*Ele + *β*_31_*DistSettl + *β*_32_*Ele*DistSettl ƒ_23_ = 0
Mod3E	Dep. Road Dist. × Ele: …the interaction of distance to road and elevation	ƒ_12_ = *β*_33_ + *β*_34_*Ele + *β*_35_*DistRoad + *β*_36_*Ele*DistRoad ƒ_13_ = *β*_37_ + *β*_38_*Ele + *β*_39_*DistRoad + *β*_40_*Ele*DistRoad ƒ_23_ = 0

*Note:* In each structure, the natural component terms ƒ_*x*_, *x* = 1, 2, or 3 represented the presence of pine martens, humans, and free‐ranging domestic cats, respectively. The occupancy models also included a detection component, whose structure was determined by comparing alternative factors that might have affected the detection process (see Table 3).

^a^ Based on the ranking in the first two steps of the multistep approach, the occupancy component of all these models also included three first‐order natural parameters: ƒ_1_ = *β*_1_ + *β*_2_*VegType + *β*_3_*Ele + *β*_4_*Ele^2^, ƒ_2_ = *β*_5_ + *β*_6_*DistRoad, and ƒ_3_ = *β*_7_ + *β*_8_*DistSettl; and a third‐order natural component: ƒ_123_ = 0.

#### 2.5.2 Activity Patterns

To compare activity patterns between pine martens and humans and between pine martens and free‐roaming domestic cats, we used a Kernel Density Estimators approach through functions available in the R packages *activity* (Rowcliffe 2022) and *overlap* (Meredith, Ridout, and Campbell 2024). Because cameras were set to take subsequent videos only after 1‐min delay, we considered the detection of a species in each recorded video (duration: 30 s) as an independent event. Before estimating the activity patterns, we adjusted the time of each record using the double‐anchoring method described in Vazquez et al. (2019) and implemented in the function *activity::solartime* to accommodate seasonal variation in sunrise and sunset times during the study period. Species‐specific 95% confidence intervals were built using a bootstrap approach that resampled 10,000 times from the data (function *activity::fitact*).

To explore similarities in activity in each of the two pairs, we estimated the three coefficients of overlap described in Ridout and Linkie (2009) and selected the most appropriate based on the number of records collected for each species (i.e., Δ_1_ when less than 50 observations are available for one of the two species; Δ_4_ when both species have at least 75 observations; Δ_5_ is never recommended; function: *overlap::overlapEst*). Following Rovero and Zimmermann (2016), we estimated the confidence intervals around each estimate of overlap by resampling the data 10,000 times using a smoothed bootstrap approach (to fill gaps in activity, e.g., for strictly unimodal species; functions *overlap::bootstrap* and *overlap::bootCI*). We selected the confidence interval values to report (out of the five returned by *overlap::bootCI*) based on whether there was bootstrap bias in the estimates, that is, a difference between the estimated overlap and the bootstrap mean, as recommended in Meredith, Ridout, and Campbell (2024). We tested the significance of the pairwise comparisons by applying the Wald test (*α* = 0.05) via *activity:compareAct*.

All data formatting and analyses were done in R (R Core Team 2022; version 4.2.1).

## 3 Results

Out of 86 initial locations, the final data sets contained data from 77 sites (for a total of 2310 active trap days), as cameras failed at nine locations mainly due to damage by wild boars and technical failures (Table S1). We detected pine martens at 55 sites, 43 of which also recorded images of both cats and humans, whereas we only detected pine marten and cats, pine marten and humans, and only pine marten at 6, 4, and 2 of these 55 sites, respectively.

### 3.1 Occupancy Modeling

**FIGURE 2:**
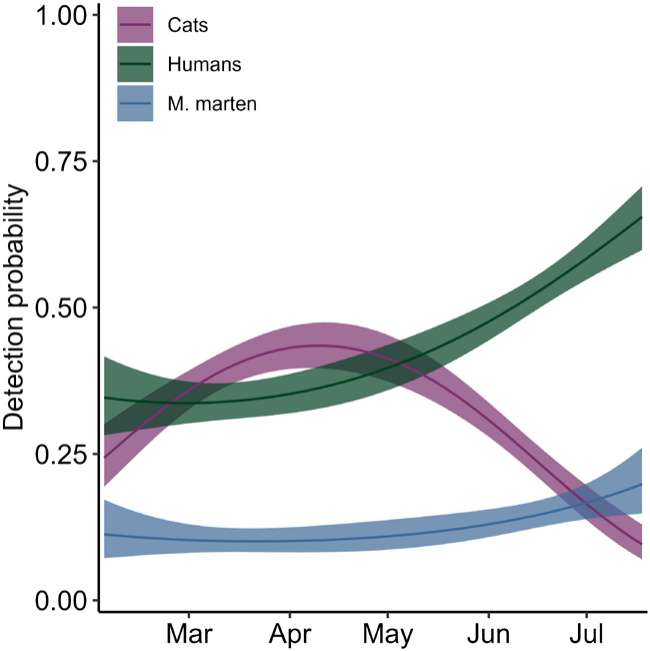
Means (solid lines) and 95% confidence intervals (shaded areas) detection probabilities for pine martens, humans, and free‐roaming domestic cats as a function of the linear and quadratic effects of Julian date, based on the most supported model structure for detection probability.

We found that daily detection probability for the three species was, by far, best described by the linear and quadratic effects of Julian date (Table 2). The probability of detecting a domestic cat showed a peak in mid‐Spring (April–May), whereas the probability of detecting a person increased throughout the period of study, with a marked rise after May. Conversely, the probability of detecting a pine marten was mostly constant between March and June and only showed a slight uptick in June–July (Figure 2). Following these results, we included linear and quadratic values of Julian date in the detection component of all the remaining occupancy models.

**TABLE 2:** Occupancy models testing the effect of different variables on detection probability.

Detection covariates	*n*Pars	ΔAIC	AICwt
Julian date + (Julian date)^2^	16	0.00	1
Julian date	13	55.63	0
Lockdown	13	102.33	0
Constant	10	180.28	0

Abbreviations: ΔAIC, AIC values in relation to the model with the lowest AIC; AIC, Akaike information criterion; AICwt, model weight; *n*Pars, number of parameters.

**FIGURE 3:**
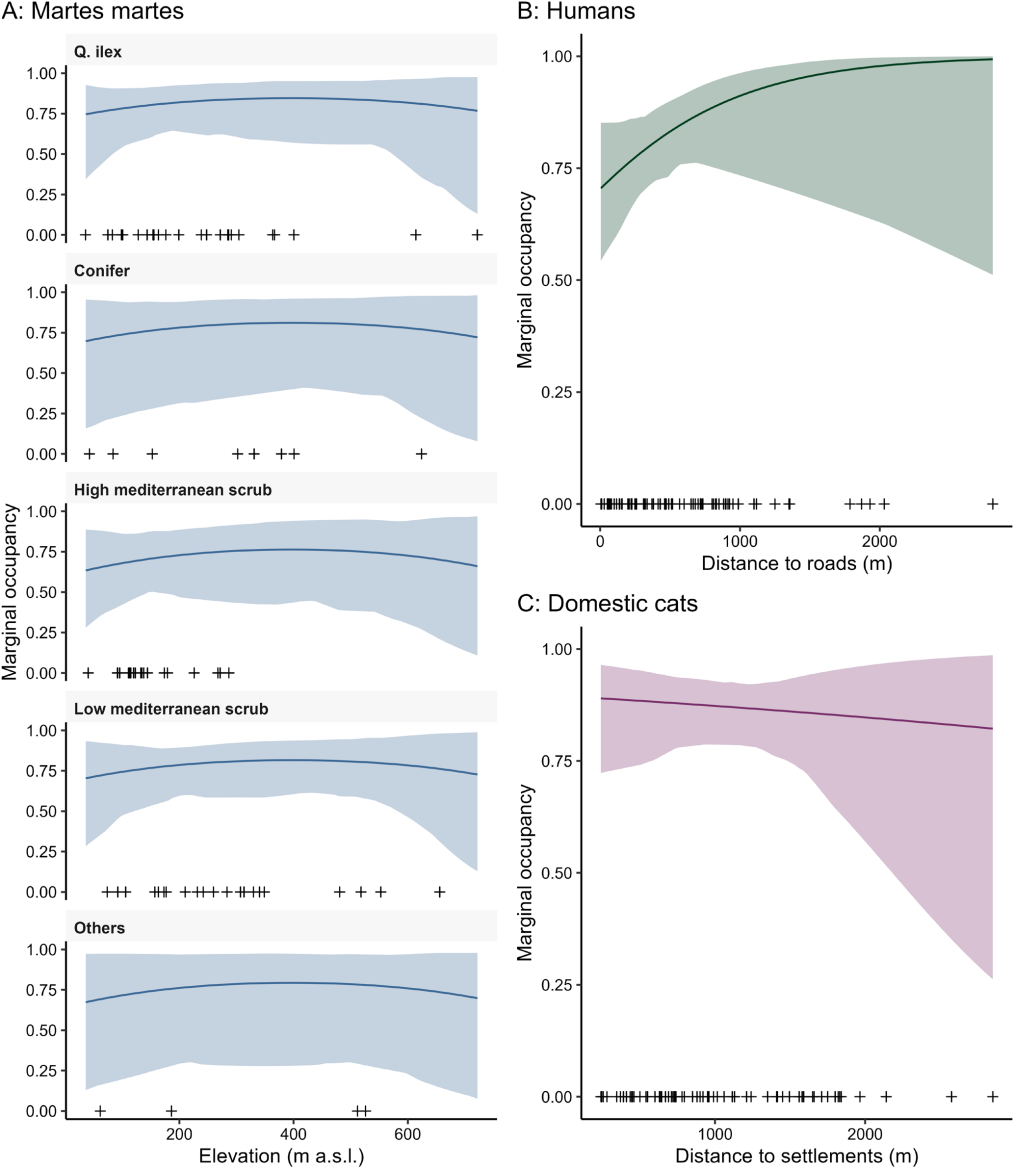
Marginal probability of occupancy for (A) *Martes martes* across vegetation types and elevation, (B) humans at increasing distances to roads, and (C) free‐roaming domestic cats at increasing distances to settlements. The crosses at the bottom of each panel represent values for the sites sampled in relation to the corresponding gradient considered. Means and 95% confidence intervals are represented by solid lines and shaded areas, respectively.

**FIGURE 4:**
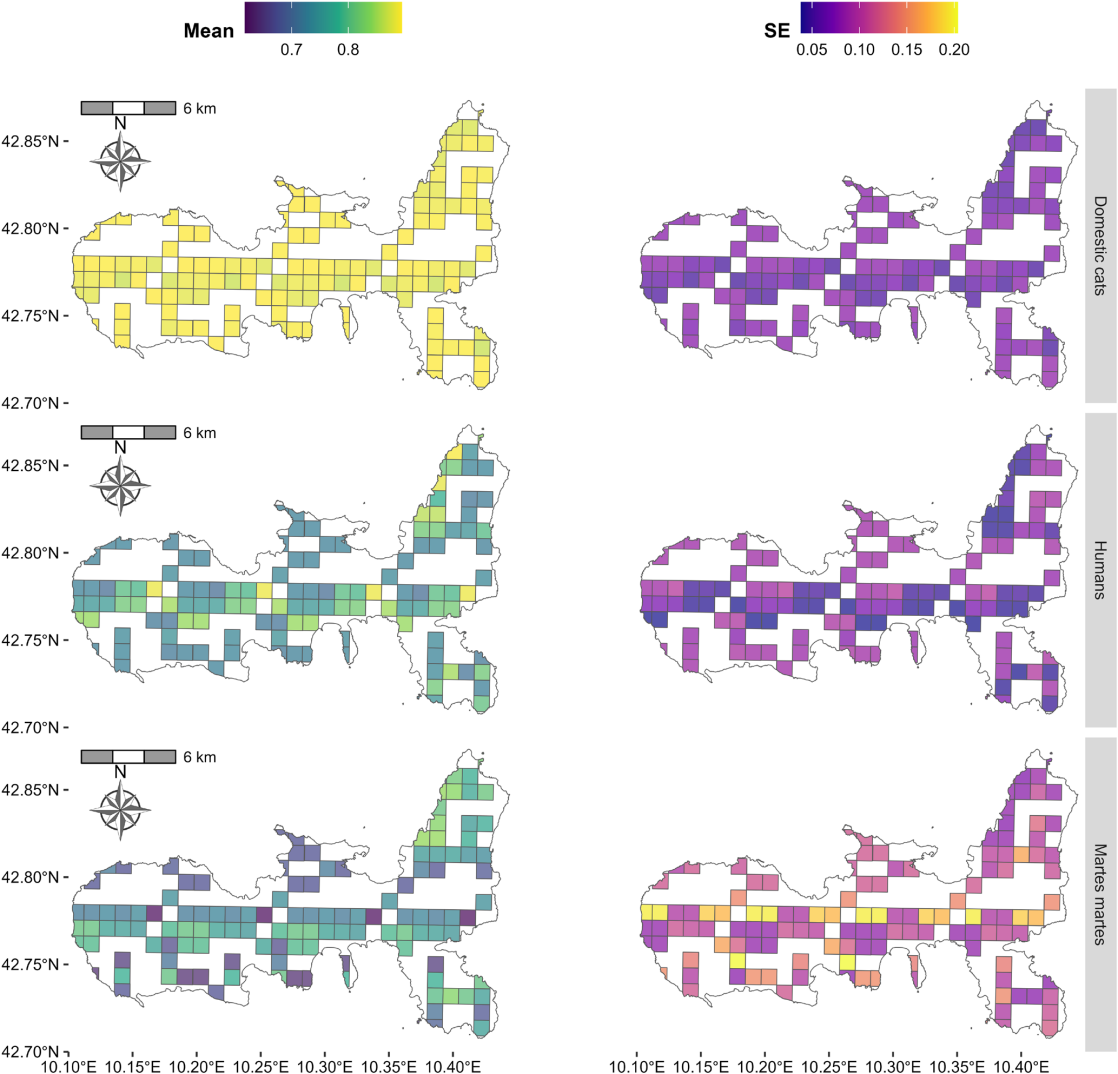
Map showing pine martens, humans, and free‐roaming domestic cats predicted occupancy probabilities across Elba Island based on the top‐ranked model. For domestic cats (top row) and humans (middle row), occupancy was modeled as a function of distance to settlements and distance to roads, respectively. Occupancy of pine martens (bottom row) was modeled as a function of vegetation types and elevation. Mean and standard error (SE) estimates are reported in the left and right panels, respectively.

When comparing the two model structures built to test the relative importance of the distance of a certain site to settlement as a descriptor of pine marten's marginal occupancy, we found that the best‐ranked model only included vegetation type and elevation as covariates. Although AIC values suggested partial support for a certain degree of association of distance to settlement to pine martens' marginal occupancy, further inspection of the values of deviance for these two models revealed that this variable had little support (i.e., it was an uninformative variable, sensu Arnold 2010; difference in deviance: 0.52). Thus, we only report results based on the top‐ranked models and retained only vegetation type and elevation as variables in the model structures fit in the third step of the occupancy analysis. The marginal probabilities of occupancy for the three species were relatively high across the variables considered important for their distribution (Figure 3). For pine marten, the mean probability of occupancy was high (≥ 0.63) at all the different altitudinal levels and vegetation types sampled, and highest in *Q. ilex* stands (≥ ~0.75; Figure 3A), with relatively high levels of uncertainty in the inner areas of the island (bottom‐left panel in Figure 4). Marginal occupancy of people increased with distance from roads, with values approaching ~1 at distance higher than 1 km (Figure 3B). As expected, marginal occupancy of domestic cats decreased at increasing distances from settlements, but only slightly, with mean estimates of probability above 0.75 even more than 2 km from human infrastructures (Figure 3C). The estimated probabilities of occupancy for people and domestic cats were consistently high to extremely high across the entire island, with very low levels of uncertainty (Figure 4). When comparing the model structures for pine marten's conditional occurrence, that is, pine martens' co‐occurrence with humans and cats, the top‐ranked model supported the hypothesis that the conditional probability of occupancy of pine martens was independent of the presence and absence of the other species (i.e., the model with all second and third‐order natural parameters set to zeros had the lowest AIC; Table 3). Although this was the best‐supported model among those included in our hypothesis‐driven model set, we report that neither vegetation type nor elevation had a statistically significant association with pine martens occupancy (i.e., all 85% and 95% confidence intervals overlapped zero; Figure S2). This result suggests that other variables not considered in this study might better describe pine marten distribution on Elba Island. We repeated this analysis using the “secondary candidate set” modeling strategy (sensu Morin et al. 2020) and obtained the same results.

**TABLE 3:** Occupancy models testing the effect of different variables on pine martens' marginal and conditional occupancy probability.

Model name^a^	Model description	*n*Pars	ΔAIC	AICwt
*Marginal occupancy* ^b^				
Mod0A^c^	Veg + Ele^2^	20	0.00	0.66
Mod0B	Veg + Ele^2^ + DistSettl	21	1.37	0.34
*Conditional occupancy*				
Mod1^c^	Indep.	20	0.00	0.57
Mod3E	Dep. Road Dist. × Ele	28	2.63	0.15
Mod3A	Dep. Road Dist.	24	3.60	0.10
Mod2	Dep. Costant	22	3.78	0.09
Mod3C	Dep. Road Dist. + Settl. Dist.	26	5.05	0.05
Mod3B	Dep. Settl. Dist.	24	6.09	0.03
Mod3D	Dep. Settl. Dist. × Ele	28	6.82	0.02

*Note:* The detection component of all the models included the linear and quadratic effects of Julian date. The single‐species occupancy components of each conditional occupancy model (i.e., the three first‐order natural parameters) were modeled as a function of the variables included in the top‐ranked marginal occupancy parameters for pine marten, and distance to roads and distance to settlements for humans and cats, respectively (see also details in Table 1). Models were ranked based on the AIC.

Abbreviations: ΔAIC, AIC values in relation to the model with the lowest AIC; AIC, Akaike information criterion; AICwt, model weight; nPars, number of parameters.

^a^ Model names match those reported in the text and in Table 1, where we report details on the structure of each model for the conditional occupancy step of the analysis.

^b^ In both marginal occupancy models, ƒ_2_ = *β*_5_ + *β*_6_*DistRoad; ƒ_3_ = *β*_7_ + *β*_8_*DistSettl; and all second‐ and third‐order natural components were set equal to zero: ƒ_12_ = ƒ_13_ = ƒ_23_ = ƒ_123_ = 0. In Mod0A, ƒ_1_ = *β*_1_ + *β*_2_*VegType + *β*_3_*Ele + *β*_4_*Ele^2^; In Mod0B, ƒ_1_ = *β*_1_ + *β*_2_*VegType + *β*_3_*Ele + *β*_4_*Ele^2^ + β_42_*DistSettl.

^c^ These models are equivalent (i.e., they have the same model structure).

### 3.2 Activity Patterns

We recorded 342, 3703, and 1062 independent encounters of pine martens, humans, and free‐roaming domestic cats, respectively, during the whole sampling period. Because all species had a number of observations higher than 75, in the next paragraphs we only report Δ_4_ as the measure of overlap (as recommended in Meredith, Ridout, and Campbell 2024; Ridout and Linkie 2009).

**FIGURE 5:**
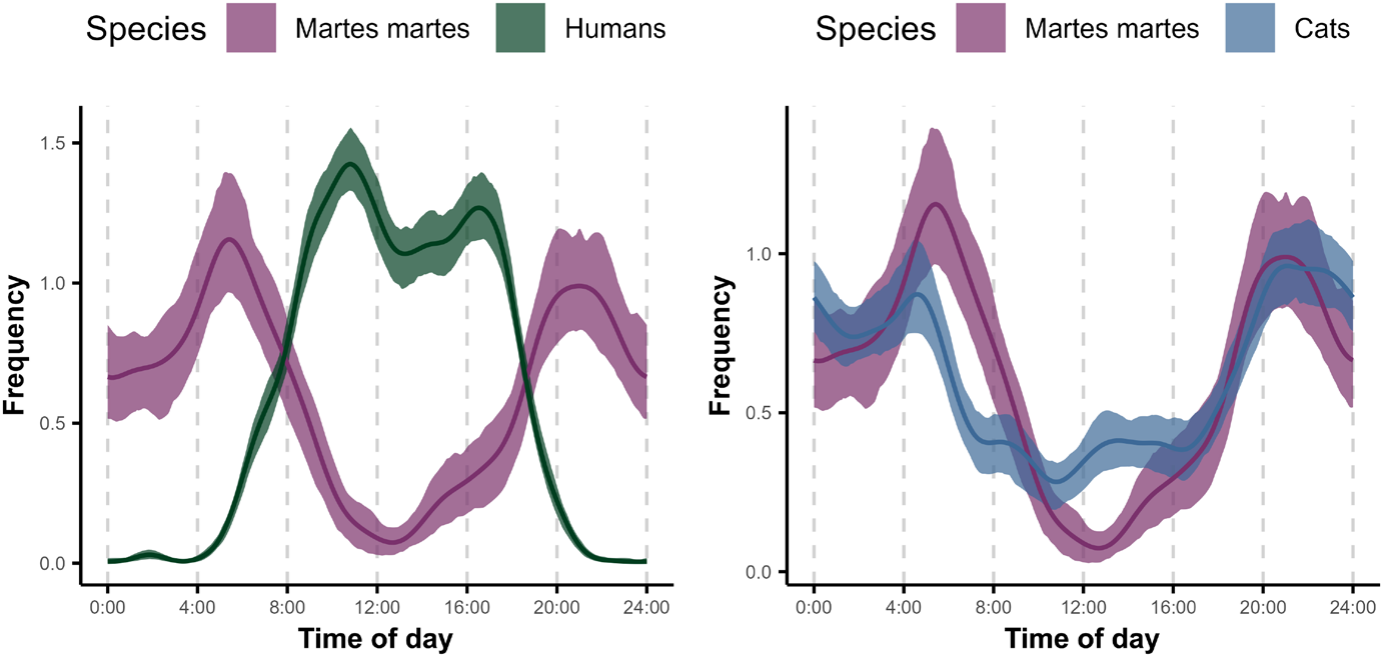
Comparison of activity patterns of pine martens and humans (left panel), and pine martens and free‐roaming domestic cats (right panel) based on circular Kernel density estimators (KDEs).

Pine martens were active mostly during the night and showed a bimodal, crepuscular activity pattern, with peaks in activity occurring between 04:00 and 06:00 and between 21:00 and 23:00 (Figure 5). Domestic cats exhibited a similar bimodal activity pattern; however, peaks in activity (centered around 4:00 and 22:00) were less marked and the level of activity during the daytime hours was higher compared to pine martens (Figure 5). Conversely, and as it might be expected, humans' diel activity occurred almost exclusively during the daytime, with the beginning and end of activity matching sunrise and sunset (Figure 5).

Pine martens and humans showed a low level of overlap (Δ_4_ = 0.31; CI adjusted for bootstrap bias: [0.27; 0.35]) and significant differences in activity patterns (*W* = 2.89; *p* value = 0.09). Despite showing a high level of overlap (Δ_4_ = 0.87; CI adjusted for bootstrap bias: [0.83; 0.92]), pine martens, and domestic cats also showed significant differences in activity (*W* = 2.53, *p* value = 0.11).

## 4 Discussion

We investigated the spatial and temporal occurrence of the European pine marten, as well as its co‐occurrence with free‐roaming domestic cats and humans on a Mediterranean island. Our results show a high level of plasticity of the European pine marten in the use of the landscape, despite the almost ubiquitous presence of the other two species, and confirm that the patterns of association of human and domestic animals' presence with wild species can be species‐ and context‐specific.

Although pine martens have been historically described as a primarily forest‐dwelling species (Brainerd and Rolstad 2002; Lindström 1989; Pulliainen 1984; Stier 2000; Storch, Lindström, and de Jounge 1990), more recent studies show that they could be defined as a tree‐dependent (Balestrieri et al. 2014; Caryl et al. 2012; Mergey, Helder, and Roeder 2011). We found probabilities of occupancy higher than 0.63 in all sampled vegetation types and elevation, suggesting that pine martens on Elba Island are limited by none of the factors considered in this study. These findings align with those of the study carried out in the Eastern portion of Elba Island, on Monte Capanne, by Mori et al. (2021). Our results thus support the hypothesis that in certain conditions the pine marten may display a more generalist attitude, as previously found by Manzo et al. (2018) in mainland Italy, at latitudes similar to those of Elba Island, and by Clevenger (1994) on Menorca Island (Western Mediterranean), where the pine marten showed no clear habitat preference. In specific contexts, such as islands, which typically feature simplified mammal communities and the absence of large predators and competitors, pine martens might be able to benefit from a large variety of environments that result in a wide variety of food resources (Vergara et al. 2016). As the only wild carnivore on Elba Island, pine martens potentially have access to a wide array of prey species. Additionally, vegetation types other than forested habitats, such as the low Mediterranean shrubland common on Elba Island, might provide the cover and resources necessary for the persistence of the species (Balestrieri et al. 2019).

Contrary to our expectation, the occupancy of pine martens was not associated with the presence of humans, even during peaks in tourism activities. Our findings differ from those obtained by Mori et al. (2021) on a portion of the same island. Their research suggested a higher mean value of pine marten occupancy probability during the winter season, possibly explained by a reduction in anthropogenic pressure. Several factors may have influenced this difference in results, including their study's original focus on larger‐bodied animals (with cameras placed higher above ground—on average 50 cm versus on average 20 cm above ground in this study—potentially leading to lower detection probability of pine martens), the period of sampling (April 2018–2019 vs. February–July 2020 in this study), and the sampling area limited only to a portion of the island (but with similar camera‐trap density). Additionally, our sampling overlapped the period of restriction to human movement imposed by the response to the COVID‐19 pandemic. Although we did not find support for differences in detection before/during/after these restrictions, the presence of humans on the island might have been lower than in the same period during other years.

It is also important to note that the statistical approach used in our study assesses the co‐occurrence of two (or more) species by directly modeling information related to human (and domestic cats') use of the sampled sites; conversely, most ecological studies so far have instead relied on proxies, such as distance to human infrastructures, to characterize the impact of human presence. In highly touristic locations, such as Elba Island, the number of people in the landscape can be highly dynamic in space and time and usually varies greatly throughout the year and within a day. In this context, proximity to human settlements or similar static covariates might be insufficient to capture the responses of wild species that dynamically adapt to instantaneous changes in their environment (Ellis‐Soto et al. 2023). In our study, the probability of people's presence approached 1 (i.e., near certainty) at distances greater than 1 km from roads, confirming the ubiquitous and high probability of human presence even in areas of the island with limited access by car, particularly in spring and summer (Figure 4). This increased human presence is primarily driven by the influx of tourists who not only visit the coastal areas but also actively explore the island's inland hiking and biking trails. Although we found that pine marten occupancy was not correlated with human presence in the study area, additional studies are needed to assess the potential effects on pine marten behavior and population density.

Comparing the patterns of activity of pine martens and humans showed a clear temporal segregation between the two species, as expected, with pine martens being mostly active at night time, as also found by Mori et al. (2021) on the Eastern portion of the Island. These findings are also consistent with previous research conducted by Oberosler et al. (2017) in the Italian Alps. Conversely, several studies carried out in other locations throughout Italy showed that pine martens tend to have cathemeral activity (Del Fante 2010; Fonda et al. 2017; Torretta et al. 2017). The temporal segregation between pine martens and humans observed in our study area could be a factor facilitating the observed spatial overlap of these two species and the apparent high tolerance toward human presence displayed by pine martens. The prevalent nocturnal patterns shown by pine martens on Elba Island could be due both to an avoidance response to humans' presence and landscape use—and the resulting reduction in the likelihood of direct interactions between the two species—and to an artifact of the sampling methods used. The camera placement strategy adopted in our study—as well as in all the other studies mentioned so far—only recorded pine marten's activity at the ground level. The species might have been active in the canopy, outside the area covered by the devices' sensors.

Our findings also supported a lack of dependence between the occurrence of free‐roaming domestic cats and pine martens. In contrast, Mori et al. (2021), based on previous studies (Balestrieri et al. 2019; Viviano et al. 2021), suggested that domestic cats (and dogs) could alter the spatiotemporal behavior of pine martens and pose a threat to the species, especially on insular systems such as Elba Island. However, the detections of cats and dogs collected by Mori et al. (2021) were too sparse to investigate any effect, as the authors themselves reported. While we also hypothesized a negative relationship of cats on pine martens' occupancy, our results do not support this hypothesis. The marginal occupancy of domestic cats in our study area was highest in the vicinity of human settlements and decreased only slightly at increasing distances, with probability of occupancy estimates above 0.75 even at distances farther than 2 km (Figures 3 and 4). This finding suggests that free‐roaming domestic cats on Elba Island are strongly associated with human settlements; however, cats are likely to also roam even in rural, isolated areas, and on the large network of trails that covers the island. This pervasive presence of cats on the island is in contrast with previous estimates of distance traveled by domestic cats; telemetry studies carried out in different parts of the world (USA, Chile, UK, Australia, and New Zealand) show that pet cats (i.e., owned indoor‐outdoor) usually stay within < 100 m of their home (Cecchetti et al. 2022; Kays, Arbogast, et al. 2020; Kays, Dunn, et al. 2020) while unconfined (i.e., free‐ranging) and unowned (i.e., feral) cats use larger areas than owned, confined (i.e., indoor‐outdoor) cats (Hervías et al. 2014; Horn et al. 2011). Cats on Elba Islands were also detected at considerable distances (up to 2.3 km) from settlements; this suggests that cats on Elba might be more likely to belong to the categories of free‐ranging and feral than indoor–outdoor cats (Cove et al. 2018). This hypothesis requires further investigation; if confirmed, it might imply important consequences on the impact of cats on wildlife on Elba Island, as cats living away from human settlements consume a higher percentage of wild prey than cats living in the proximity of settlements (Cove et al. 2018). To our knowledge, this is the first estimate of the occupancy of domestic cats across Elba Island and might provide important guidance for future conservation actions in the PNAT.

The analysis of activity rhythms between pine martens and domestic cats exhibited similar bimodal patterns, with notable differences. The timing of the peaks in activity was similar in the two species (both active at night time), but their intensity was less pronounced in cats than in pine martens. The activity levels of pine martens during the daytime were low compared to the levels in domestic cats, suggesting a period of reduced ground‐level activity or rest for the mustelid. Despite these differences, the analysis revealed a high, but not significant, level of overlap in activity between pine martens and domestic cats (overlap coefficient: Δ_4_ = 0.87; *p* value = 0.11), indicating extended periods when both species were active simultaneously but with noticeable differences in their activity rhythms. The higher level of daytime activity observed in domestic cats may be attributed to their domestication and adaptation to human routines, as they often align their activity with human presence and daily schedules (Horn et al. 2011; Piccione et al. 2013), while the patterns observed in pine martens might be driven by other factors (as suggested above). The absence of association of domestic cats with pine marten distribution suggests that cats might not act as strong competitors or predators limiting pine martens' occupancy on the island, and this might contribute to the pine marten's status as the apex predator on Elba. Without significant competition for resources, the pine marten population may thrive and potentially reach high densities (Breault et al. 2021; Crooks and Soulé 1999; Prugh et al. 2009).

Whereas our results do not support the hypothesis of a negative effect of humans and domestic cats' presence on pine martens' occupancy and activities, there are important aspects to consider when interpreting these findings. Humans and cats might impact pine martens' densities and fitness, as well as the availability of the resources that the species requires, through other mechanisms. Additionally, occupancy might be a population metric too coarse to detect changes in pine marten population abundance associated with the presence of the other species, and our sample size might not have been large enough to reveal significant effects, especially if those were small. Additionally, evidence of statistical interactions among species might not correspond to ecological interactions: species might co‐occur in space and/or time driven by factors other than interspecies ecological processes such as competition, mutualism, and predation (Blanchet, Cazelles, and Gravel 2020; Dormann et al. 2018). Further research and investigations into the population dynamics, predator–prey relationships, and resource availability on Elba Island and other insular and continental areas would be valuable to gain a more comprehensive understanding of the factors influencing the pine marten population dynamics and its role as the apex predator.

## 5 Conclusion

We found no evidence of the impact of human presence driven by tourism on pine martens distribution on Elba Island, despite the surge in people presence occurring on the island from winter to summer—our sampling period—when the island's population increases from 32,000 to 300,000 inhabitants, with approximately 1,800,000 tourists per year (according to data from Regione Toscana). Similarly, we found no association between the distribution of domestic cats and pine martens. However, given that cats are one of the major threats to biodiversity (Loss, Will, and Marra 2013; Mori et al. 2019; Trouwborst, McCormack, and Martínez Camacho 2020), targeted studies are needed to assess their impact on other species inhabiting Elba Island. Importantly, the findings of this study might be limited to the specific context of Elba Island and to the context in which the data were collected, as the absence of disturbance by cats' and humans' presence and the absence of other predators may be unique to this particular system. Conclusions may differ in other ecosystems or regions where different predator–prey dynamics or species interactions exist. Our findings confirm that the negative effects of the presence of humans and cats might not be ubiquitous, as commonly hypothesized, and species' responses might be driven by local contexts and species characteristics (Tucker et al. 2023).

Our study is an example of how camera traps could be a valuable tool for studying the impact of tourism on species of conservation concern, particularly in areas of conservation importance such as Elba Island, where a significant portion of the territory is protected under the Parco Nazionale dell'Arcipelago Toscano. Similar studies in other contexts could help understand and quantify directly the response of wild species to the increased and pervasive presence of people in natural areas and help to better manage and regulate access to recreational activities in space and time.

## Author Contributions

**Emiliano Manzo:** conceptualization (lead), data curation (lead), investigation (lead), methodology (lead), project administration (lead), supervision (lead), visualization (equal), writing – original draft (lead). **Fabiola Iannarilli:** conceptualization (lead), data curation (lead), formal analysis (lead), methodology (lead), visualization (lead), writing – original draft (lead). **Filippo Dell'Agnello:** conceptualization (equal), data curation (equal), investigation (equal), methodology (equal), writing – review and editing (equal). **Paola Bartolommei:** conceptualization (equal), data curation (equal), investigation (equal), methodology (equal), writing – review and editing (equal). **Andrea Bonacchi:** data curation (equal), methodology (equal), writing – review and editing (equal). **Stefania Gasperini:** data curation (equal), methodology (equal), writing – review and editing (equal). **Roberto Cozzolino:** conceptualization (equal), funding acquisition (lead), project administration (equal), supervision (equal), writing – review and editing (equal).

## Conflicts of Interest

The authors declare no conflicts of interest.

## Supporting information


Appendix S1.

## Data Availability

The data and code that support the findings of this study are openly available in the repository named “Data, R Code, and Output Supporting: Assessing the co‐occurrence of European pine marten ( *Martes martes* ) with humans and domestic cats on a Mediterranean island” at https://doi.org/10.5061/dryad.n02v6wx0m.
