# Supplementary material for: Assessing the Co‐Occurrence of European Pine Marten (Martes martes) With Humans and Domestic Cats on a Mediterranean Island
Source: Ecol Evol. 2024 Nov 29;14(12):e70651. doi: 10.1002/ece3.70651 (PMC11606699; doi:10.1002/ece3.70651)
Supplement: Supplementary file 1 — Appendix S1. [file ECE3-14-e70651-s001.docx]

## Appendix


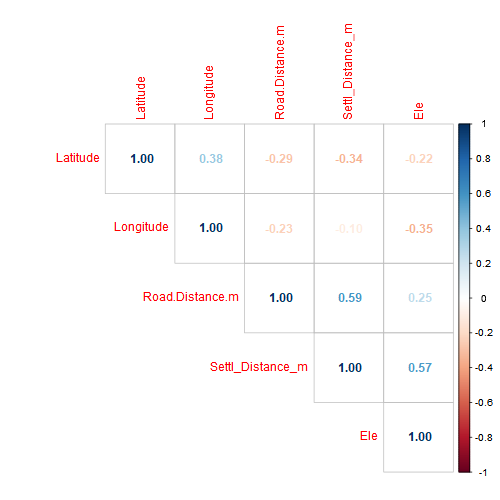


Figure S1. Correlation among the site-level covariates included in the models.


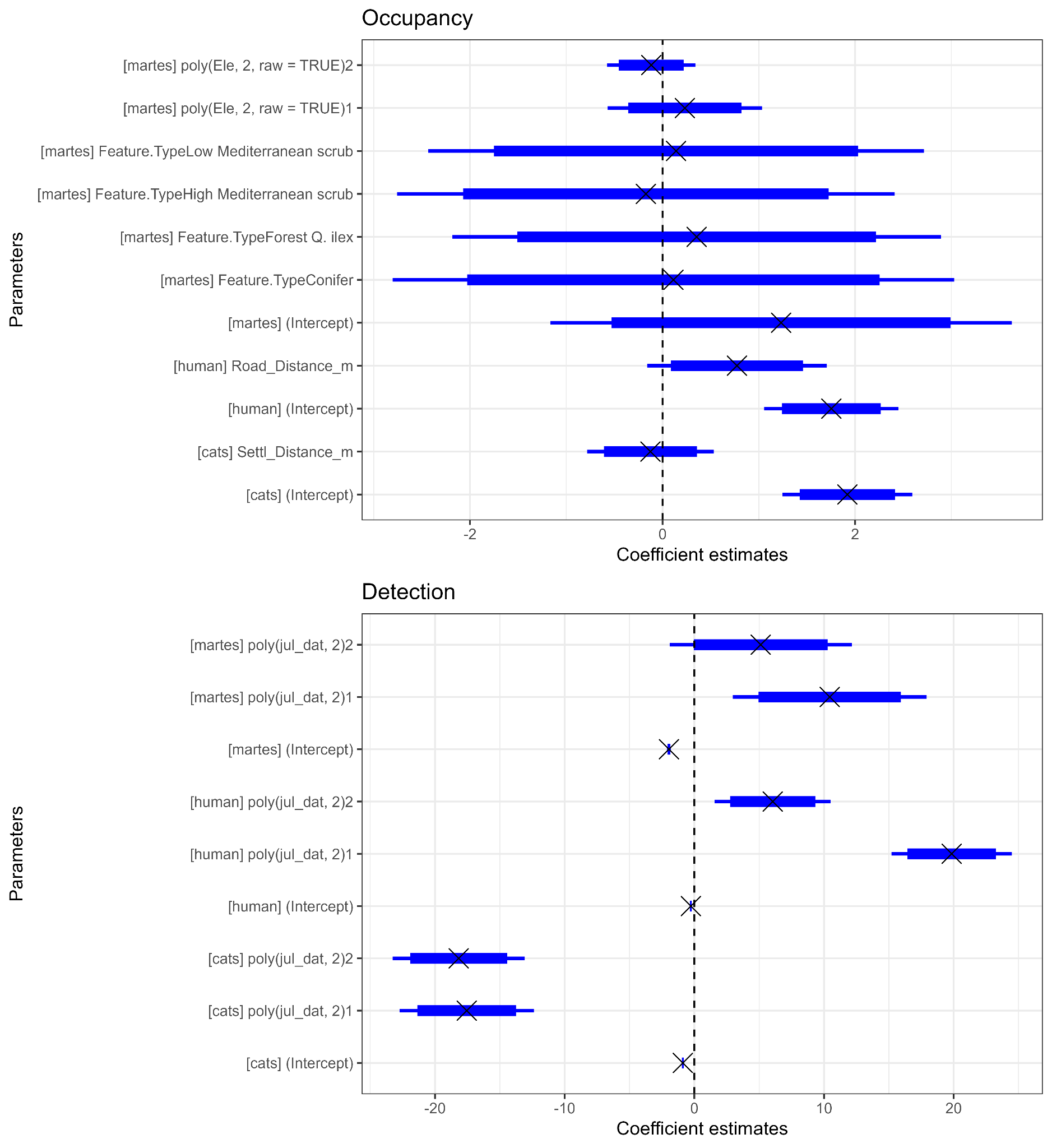


Figure S2. Coefficient estimates for the top ranked model (Mod1 in Tables 1 and 3). Thicker and thinner bars represent 85 and 95% confidence intervals; crosses represent mean estimates.

**Table S1**. Survey specification for each sampling period. The table reports the total number of damaged camera traps (CTs), the total number of working cameras at the end of the sampling periods, and sampling effort as the total number of active camera days during each sampling period.

| Sampling Period | Damaged CTs | Working CTs | Sampling Effort |
| --- | --- | --- | --- |
| February | 5 | 18 | 540 |
| March | 2 | 20 | 600 |
| May | 0 | 20 | 600 |
| June | 2 | 19 | 570 |
